# Electrocardiogram ST-Segment Morphology Delineation Method Using Orthogonal Transformations

**DOI:** 10.1371/journal.pone.0148814

**Published:** 2016-02-10

**Authors:** Miha Amon, Franc Jager

**Affiliations:** Faculty of Computer and Information Science, University of Ljubljana, Večna pot 113, 1000 Ljubljana, Slovenia; Ulm University, GERMANY

## Abstract

Differentiation between ischaemic and non-ischaemic transient ST segment events of long term ambulatory electrocardiograms is a persisting weakness in present ischaemia detection systems. Traditional ST segment level measuring is not a sufficiently precise technique due to the single point of measurement and severe noise which is often present. We developed a robust noise resistant orthogonal-transformation based delineation method, which allows tracing the shape of transient ST segment morphology changes from the entire ST segment in terms of diagnostic and morphologic feature-vector time series, and also allows further analysis. For these purposes, we developed a new Legendre Polynomials based Transformation (LPT) of ST segment. Its basis functions have similar shapes to typical transient changes of ST segment morphology categories during myocardial ischaemia (level, slope and scooping), thus providing direct insight into the types of time domain morphology changes through the LPT feature-vector space. We also generated new Karhunen and Lo ève Transformation (KLT) ST segment basis functions using a robust covariance matrix constructed from the ST segment pattern vectors derived from the Long Term ST Database (LTST DB). As for the delineation of significant transient ischaemic and non-ischaemic ST segment episodes, we present a study on the representation of transient ST segment morphology categories, and an evaluation study on the classification power of the KLT- and LPT-based feature vectors to classify between ischaemic and non-ischaemic ST segment episodes of the LTST DB. Classification accuracy using the KLT and LPT feature vectors was 90% and 82%, respectively, when using the *k*-Nearest Neighbors (*k* = 3) classifier and 10-fold cross-validation. New sets of feature-vector time series for both transformations were derived for the records of the LTST DB which is freely available on the PhysioNet website and were contributed to the LTST DB. The KLT and LPT present new possibilities for human-expert diagnostics, and for automated ischaemia detection.

## Introduction

**Fig 1:**
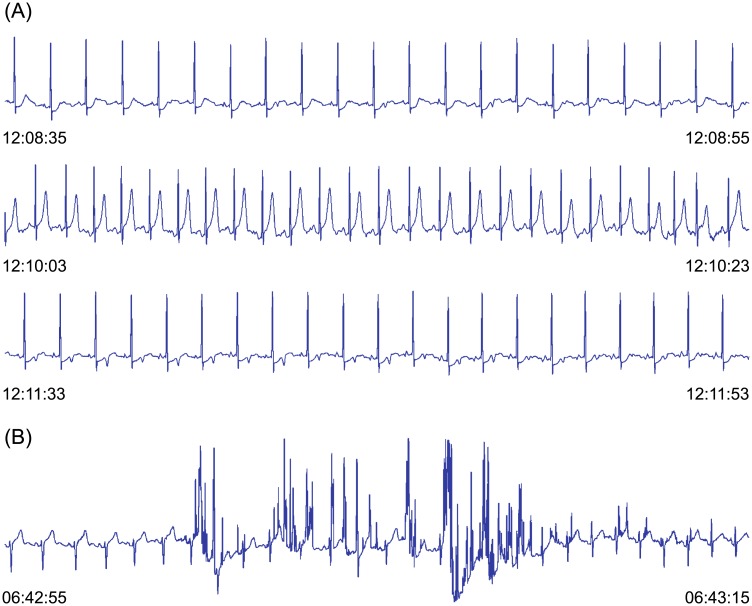
Typical data segments of AECG records. (A) The beginning, extrema and end (from top to bottom) of a transient ST segment episode compatible with ischaemia. (B) Severe noise.

Ambulatory electrocardiogram (AECG) monitoring of long term electrocardiogram (ECG) records, obtained during the patient’s normal daily activities, is important in the assessment of symptomatic and asymptomatic, or “silent”, ischaemia which may lead to myocardial infarction and consequently death. Due to the long duration of records (24 hours), which means an enormous amount of data, and due to the possible presence of severe noise, automated procedures for the extraction of diagnostic and morphologic features are becoming very important. A convenient method for the representation and tracking of transient signal-shape changes are time series of features. Automated analysis is of great help to clinicians in early assessment of cardiac ischaemia severity for the accurate interpretation of relevant clinical results and for proper treatment of the patient. Fig 1 shows two typical data segments of AECG records. A *transient ischaemic ST segment episode* compatible with ischaemia is present in the upper data segment (Fig 1A). An increased heart rate and transient morphology change of the ST segments of heart beats may be observed. The lower data segment (Fig 1B) is an example of severe noise which is often present in AECG records and cause the main problems during the visual and automatic assessing of the severity of ischaemic ST segment episodes.

**Fig 2:**
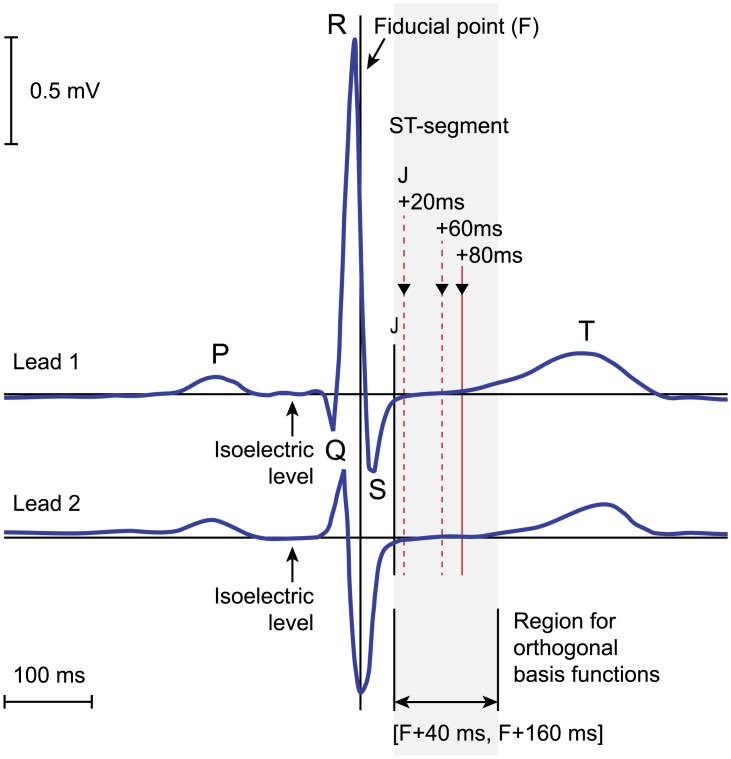
The ECG of a normal heartbeat. A heartbeat of a two-lead AECG record with marked points and intervals to estimate ST segment diagnostic and morphologic features.

Long term AECG records typically show *significant* (> 50*μ*V) transient changes in the amplitude of the ST segment level, and transient changes of ST segment morphology, forming transient ST segment episodes. ST segment level is measured 80 ms, or 60 ms, if the heart rate exceeds 120 bpm, after the end of ventricle depolarization period, i.e., 80 ms, or 60 ms, after point J in the ECG (see Fig 2). These significant transient changes are caused by ischaemia, which is of main clinical importance, and by a variety of reasons other than ischaemia. Significant non-ischaemic ST segment changes may be: (1) *slow drifts* of the ST segment level due to slow diurnal changes or effects of medication, or due to non-postural changes in the cardiac electrical axis; (2) sudden shifts of the ST segment level due to shifts of the mean cardiac electrical axis of the heart (*axis shifts*) as a consequence of postural changes or sudden changes in ventricular conduction [1, 2]. Another problem are *transient non-ischaemic heart-rate related ST segment episodes* appearing as transient changes of the ST segment level, but they are actually due to increased heart rate and shortening of RR intervals, and, e.g., consequently moving of the T wave closer to the QRS complex, thus distorting ST segment level measurements. These episodes are not caused by an obstruction of blood flow to the heart. Reliable automated ST analyzers should be able to distinguish clinically significant ischaemic ST changes from non-ischaemic ones.

An early study on the visual examination of ECG variables plotted in high resolution temporal trend format for the retrospective identification of the beginnings and ends of ST episodes [3] proved the technique to be superior (sensitivity of 100%, positive predictivity of 100%) to the conventional visual scrutiny of raw ECG signals (sensitivity of 82.5%, positive predictivity of 95.7%) and suitable for the quantification of ST episodes. Due to the enormous amount of data in AECG records (24 hours), and due to severe high frequency muscle noise and outliers, which are usually present and complicate manual analysis, automated techniques to estimate diagnostic and morphologic features are necessary. A key difficulty is that traditional ST segment level measuring in a single point at a fixed location (J + 80(60) ms) is not an adequately precise technique for real ischaemic ST episodes detection.

The use of orthogonal transformations is important. Orthogonal transformations reduce the dimensionality of data, retain information related to useful signal, and represent the data in terms of uncorrelated features (sets of coefficients or feature vectors) that are based on orthogonal basis functions. The Karhunen and Loève Transformation (KLT) yields minimum expected least mean squared error on the reconstruction of pattern vectors. The purpose so far of using the KLT in ECG signal analysis was noise estimation [4], visually identifying acute ischaemic episodes [5], the representation of ECG morphology [4, 6], the automated detection of transient ST segment episodes during AECG monitoring [7, 8], the analysis of the cardiac repolarization period (ST-T complex) [9–12], visually identifying and manually annotating the transient ischaemic and non-ischaemic ST segment episodes of the LTST DB [1], and automated ischaemic and non-ischaemic heartbeat classification [13]. The Hermite polynomials were used for estimating ECG wave features [14] and for clustering ECG complexes [15]. The Hermite, Legendre and Chebyshev polynomials were used for filtering and representing ECG morphology [16]. The feasibility of ECG feature extraction and representation of transient ST segment morphology of mice ECG using the Chebyshev-polynomial based transformation was shown [17]. Furthermore, combinations of the KLT, Legendre polynomials and a variety of other ECG features were used for transient ischaemic and non-ischaemic ST episode classification [18–20].

Robust methods for parameter extraction for use in intensive care units are becoming important [21]. The severe noise frequently present in AECGs necessitates the development of robust noise resistant delineation systems to accurately trace transient changes of ST segment morphology during myocardial ischemia in terms of diagnostic and morphologic feature-vector time series. Robust parameter extraction techniques yield nearly the same performance no matter which records are analyzed.

In this paper, we present a new robust delineation method for ST segment morphology feature extraction, and transient ST segment morphology-change representation and tracing in the sense of generation of ST segment diagnostic and morphologic feature-vector time series using orthogonal transformations. We present a new approach for shape representation of transient ST segment morphology changes using the orthogonal Legendre polynomials, i.e., Legendre Polynomial based Transformation (LPT) of ST segment. Furthermore, we develop new ST segment KLT basis functions derived from a robustly generated covariance matrix composed of the ST segment pattern vectors of the entire LTST DB. We then assess the representational power of the KLT- and LPT-based derivation of morphology feature-vector time series through a study on the representation of significant transient ischaemic and non-ischaemic ST segment morphology categories. We also evaluate the classification power of the KLT- and LPT-based feature vectors to distinguish between the ischaemic and non-ischaemic ST segment episodes of the LTST DB.

## Methods

Fig 2 shows the ECG of a normal heartbeat with marked points and intervals to estimate the ST segment diagnostic and morphologic features that are relevant to represent, monitor and characterize transient ischaemic and non-ischaemic ST segment changes. The diagnostic ST segment feature, like ST segment level, provides direct measurement of raw ST segment pattern vectors in time domain in a single point at a fixed location (J + 80(60) ms), while orthogonal transformation-based ST segment morphologic feature vectors utilize information from the entire ST segment, thus providing high representational power in terms of ST segment morphology categories, as well as subtle morphology features, and differentiation between transient ischaemic and non-ischaemic ST segment events.

**Fig 3:**
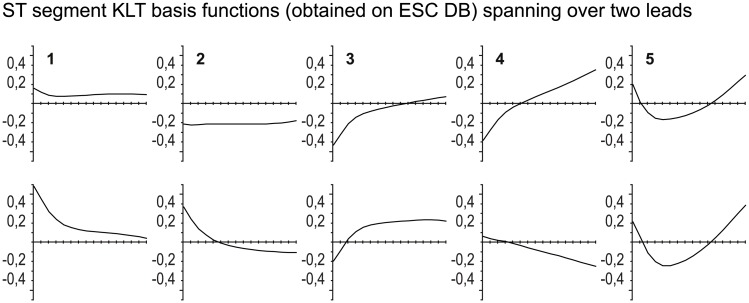
The ST segment KLT basis functions (they span over two ECG leads) obtained from the ESC DB. The duration of the ST segment basis functions is 2 x 120 ms, from *F* + 40 ms to *F* + 160 ms, in 2 x 16 sample resolution.

The motivation for a new approach using the orthogonal transformation of ST segment based on orthogonal polynomials comes from observing the shapes of the ST segment KLT basis functions [7] obtained from the *European Society of Cardiology ST-T Database* (ESC DB) [2, 22], the standard reference for assessing the quality of AECG analyzers. These basis functions (see Fig 3) span over two ECG leads. We found that the shapes of the KLT basis functions are similar to the three main morphologic categories of ST segment morphology changes. The first and second KLT basis functions have shapes similar to a constant function and thus correspond to the elevation or depression of ST segments. The third and fourth KLT basis functions have shapes similar to a linear function and thus correspond to the slope of ST segments. The fifth (and sixth) KLT basis function has a shape similar to a quadratic function and thus corresponds to the scooping of ST segments. However, for these basis functions there is no natural mapping between the deflections of ST segment KLT feature-vector time series and the actual deflections of ST segment morphology change categories like: depression/elevation, up- and down-sloping, and scooping.

**Fig 4:**
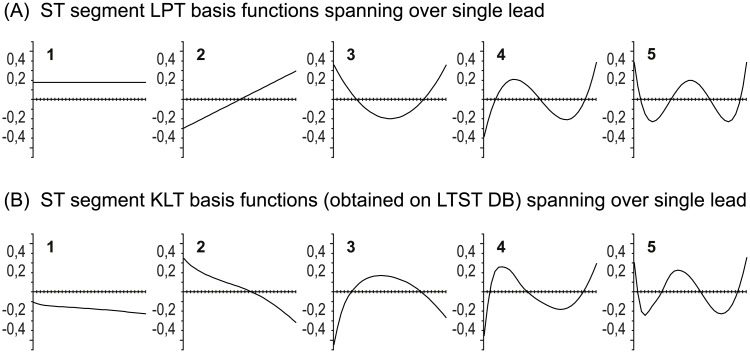
The derived LPT and KLT basis functions. (A) The Legendre orthogonal polynomials as the ST segment basis functions, **Φ**_L_, spanning over a single ECG lead. (B) The ST segment KLT basis functions, **Φ**_K_, obtained from the LTST DB which span over a single ECG lead. The duration of the ST segment basis functions is 120 ms, from *F* + 40 ms to *F* + 160 ms, and are in 32 sample resolution.

Therefore, we concluded to devise a set of new orthogonal basis functions that span over a single ECG lead, with similar characteristics as the KLT, and with the further advantage of strict correspondence to elevation/depression, slope change, and scooping, to better delineate the transient shapes of ST segment morphology changes. The first three Legendre polynomials (a constant, linear function and square function) uniquely possess these shapes and they are also orthogonal. We used them to derive a new set of basis functions for the Legendre Polynomial-based Transformation of ST segment (Fig 4A) that span over a single ECG lead.

In addition, we derived a new set of ST segment KLT basis functions, which also span over a single ECG lead, from the entire collection of the records of the LTST DB using a robust covariance matrix, with outliers due to noise rejected. The LTST DB database contains approximately a ten times larger ECG data set compared to the ESC DB database, covers a considerably greater amount of “real-world” data, and spans a wide variety of significant ischaemic and non-ischaemic ST segment episodes and other ST segment morphology change events due to axis shifts and conduction changes. The shapes of the newly derived KLT basis functions (Fig 4B) are more similar to typical morphology shape changes of ST segments, very similar to the LPT basis functions, and allow more accurate single-lead tracing of ST segment morphology change categories.

### The Legendre Polynomial based Transformation of ST segment and derivation of the LPT basis functions

The Legendre polynomials [23] are a class of orthogonal polynomials. They are solutions to the Legendre differential equation. The first five Legendre polynomials are:

$$\begin{array}{c} P_{0}\left(x\right)=1, \end{array}$$ (1)

$$\begin{array}{c} P_{1}\left(x\right)=x, \end{array}$$ (2)

$$\begin{array}{c} P_{2}\left(x\right)=\frac{1}{2}\left(3x^{2}-1\right), \end{array}$$ (3)

$$\begin{array}{c} P_{3}\left(x\right)=\frac{1}{2}\left(5x^{3}-3x\right), \end{array}$$ (4)

$$\begin{array}{c} P_{4}\left(x\right)=\frac{1}{8}\left(35x^{4}-30x^{2}+3\right). \end{array}$$ (5)

The Legendre polynomials can be generated by the following recurrence relation:

$$\left(j+1\right)\;P_{j+1}\left(x\right)\;-\;\left(2j+1\right)\;x\;P_{j}\left(x\right)\;+\;j\;P_{j-1}\left(x\right)\;\;=\;\;0\;,\;\;\;\;\;j=1,2,3,...\;.$$ (6)

They are orthogonal over the range [-1,1] satisfying the orthogonality relationship:

$$\int_{-1}^{1}\;P_{n}\left(x\right)\;P_{m}\left(x\right)\;dx\;\;=\;\;\frac{2}{2n-1}\;\delta_{mn}\;,\;\;\;\;\;n=1,2,...\;,\;\;\;\;\;m=1,2,...\;,$$ (7)

where *δ*_*mn*_ is the Kronecker delta. The Legendre polynomials possess the desired orthogonality and desired shapes. Orthogonality is an important property for basis functions to be uncorrelated, thus preventing information scattering among different axes of the transformed space and enabling transformation reversibility and the derivation of residual errors. The shapes of the first three Legendre polynomials (a constant, linear function and square function) have the advantage of direct insight into the most important morphological changes of ST segments in time domain (elevation or depression, positive or negative slope, and positive or negative scooping) through the feature space, if the polynomials are used as transformation basis functions. Such a similarity in terms of polynomial shapes can also be observed if considering some other classes of orthogonal polynomials like Chebyshev polynomials [17], however the Chebyshev polynomials loose the desired shapes (a constant, linear function and square function) in their orthogonal form.

The Legendre polynomials can also be generated by the Gram-Schmidt orthonormalization [24] to functions of the following form:

$$Q_{j-1}\;\left(x\right)\;\;=\;\;x^{\;j-1}\;,\;\;\;\;\;j=1,2,3,...\;,$$ (8)

on the interval [-1,1] with respect to the weighting function, *w*(*x*) = 1. In order to derive the discrete basis functions for the orthonormal transformation of the ST segment based on the Legendre polynomials, we first constructed a discrete matrix, **Ω**(_*ij*_), which is composed from the polynomials *Q*_*j*−1_, sampled at *M* = 32 points in the range [-1,1]:

$$\Omega_{\left(ij\right)}\;=\;Q_{j-1}\;\left[\;2\;\;\frac{\left(i-1\right)}{\left(M-1\right)}\;-\;1\right]\;,\;\;\;\;\;i=1,2,...,M,\;\;j=1,2,...,M\;,$$ (9)

where [.] denotes the argument, *x*, of the polynomials from the range [-1,1], *i* denotes the sample number of discretized polynomials, and *j* is the polynomial number. The discrete Gram-Schmidt orthonormalization [25] to the matrix **Ω**(_*ij*_) generates a matrix, **Φ**(_*ij*_), of the same dimensionality *M* × *M*, composed from orthonormal discretized Legendre polynomials. During the Gram-Schmidt process, iteratively derived orthogonal polynomials, **Ψ**(_*ij*_), are normalized, yielding the matrix **Φ**(_*ij*_):

$$\Phi_{\left(ij\right)}\;=\;\;\frac{\psi_{ij}}{\sqrt{\sum_{k=1}^{M}(w_{k}\;\psi_{kj}^{2})}}\;,\;\;\;\;\;i=1,2,...,M,\;\;j=1,2,...,M\;,$$ (10)

where *ψ*_*ij*_ are the elements of the discretized orthogonal Legendre polynomials of the matrix **Ψ**(_*ij*_), and *w*_*i*_ = 1 is the discretized weighting function, *w*(*x*) = 1. Fig 4A shows the first few orthonormal LPT basis functions of the matrix **Φ**_L_ = **Φ**(_*ij*_), as the order of the polynomials increases.

Due to discretization and a degree of numerical instability generally present in numerical algorithms, some loss of orthogonality in the generated basis functions is expected [25]. (Note that this is also true for the discrete KLT.) The LPT basis functions contained in the **Φ**_L_ matrix are expected to be orthonormal,

$$\Phi_{L}\;\Phi_{L}^{T}\;\;=\;\;I\;,$$ (11)

where **I** is the identity matrix. This holds to an adequately high degree of numerical accuracy. If the discrete Gram-Schmidt orthonormalization is applied to the polynomials *Q*_*j*−1_, the result are orthonormal discretized Legendre polynomials which only slightly differ from their continuous analogue [25]. Due to the iterative nature of the generation algorithm, numerical errors grow with the increasing basis function number. We tested the orthonormality of the discrete LPT basis functions derived by calculating the values of elements of the identity matrix. For the first 10 LPT basis functions of the matrix **Φ**_L_, the maximal numerical error of the diagonal elements of the identity matrix, **I**, was 3,4 . 10^−6^, and for the off-diagonal elements it was 1, 0 . 10^−5^.

The LPT expansion is thus based on mutually orthogonal Legendre polynomials used as the basis functions for the transformation. Since the Legendre polynomials were chosen intentionally due to their shape similarity to the KLT basis functions in the descending order of the associated eigenvalues, it can be reasoned that the Legendre polynomial based expansion contains most of the morphology information in the first few axes of the new coordinate system as well.

### The Karhunen and Loève Transformation and the derivation of new KLT basis functions

The KLT expansion is based on mutually orthogonal eigenvectors belonging to eigenvalues in descending order of the covariance matrix associated with the pattern vectors. It is possible to approximate the pattern vector with least mean square error through a feature vector of reduced dimension in comparison to other suboptimal transformations. Detailes about the KLT can be found elsewhere [26, 27]. To avoid the problem of sensitivity of eigenvectors to noise pattern vectors, we used the kernel-approximation method [28] by which we rejected noisy outliers and replaced pattern classes by their means yielding a robust covariance matrix [28].

To construct the robust covariance matrix, we used clean heart beats from the LTST DB left after preprocessing the records with robust KLT feature-space based noise and the outlier extraction procedure [7]. The procedure proved to be robust and accurate. On average, 8.51% of heart beats were rejected from each of the LTST DB records.

Then we attached the first and second lead of 86 total records from the LTST DB one after the other. The latter is justified since single lead basis functions independent of the actual physiological ECG lead are desired. Besides, physiological ECG leads are not consistently mapped to the same lead number in the records of the LTST DB. Thus we got 15,661,886 total pattern vectors from 7,830,943 clean heart beats from the database. Input pattern vectors of deviating intervals (ischaemic, non-ischaemic) and of intervals with no deviation of the records of the LTST DB were separated to form classes, which were then replaced by their means, and centralized by subtracting the mean vector obtained over all classes, thus forming a robust covariance matrix. There were 1642 ischaemic intervals, 510 non-ischaemic and 2298 intervals with no deviation. Fig 4B shows the first five newly derived ST segment KLT basis functions, **Φ**_K_, in the descending order of magnitude of their corresponding eigenvalues. Note the similarity between the KLT and LPT basis functions.

### Derivation of ST segment diagnostic and morphologic feature-vector time series

The developed delineation method includes a preprocessing step, the derivation of traditional time domain diagnostic features, derivation the KLT- and LPT-based ST segment feature vectors, and construction of the feature-vector time series. The preprocessing step includes following essential tasks: heartbeat detection and classification, estimation of stable fiducial point, estimation of the iso-electric level for each heartbeat, noise removal, and removal of abnormal heart beats and their neighbors. In the preprocessing step, we applied: Aristotle arrhythmia detector [29] detecting and classifying heart beats, and estimating the stable fiducial point, an algorithm that looks for the PQ interval as the “most flat” signal interval prior the heartbeat’s fiducial point and estimates the iso-electric level [30], removal of high-frequency noise using a 6-pole low-pass Butterworth filter with the cut-off frequency at 55Hz, removal of baseline wander using cubic spline approximation and subtraction technique, and removal of abnormal heart beats and their neighbors.

#### Derivation of ST segment diagnostic feature-vector time series

Derivation of traditional time domain ST segment diagnostic feature-vector time series is an essential part of delineating transient ST segment morphology changes. On the other hand, we want to study and emphasize the representational power of the KLT- and LPT-based ST segment morphologic feature-vector time series in comparison to traditional time domain diagnostic feature-vector time series. With the proposed delineation method, time domain ST segment diagnostic feature-vector time series of instantaneous heart rate, ST segment level, and ST segment slope are derived.

Instantaneous heart rate, *h*(*j*), where *j* denotes the heartbeat number, is defined by consecutive measurements of RR intervals between heart beats. A sample of the ST segment level time series, *s*_l_(*i*, *j*), where *i* denotes the lead number, is defined as:

$$s_{l}\left(i,j\right)\;\;=\;\;a_{80(60)}\left(i,j\right)\;\;-\;\;z\left(i,j\right)\;\;,$$ (12)

where *a*_80(60)_(*i*, *j*) is the ST segment amplitude of the *j*-th heartbeat, estimating the ST segment amplitude at the point J+80(60) ms, and *z*(*i*, *j*) is its iso-electric level. The point of measurement of the ST segment amplitude, *a*_80(60)_(*i*, *j*), is linearly adjusted between the points *F*(*i*, *j*) + 160 ms and *F*(*i*, *j*) + 120 ms as the heart rate *h*(*j*) varies between 120 bpm down to 100 bpm. The value of the ST segment slope, *s*_*s*_(*i*, *j*), is estimated simply as the amplitude difference:

$$s_{s}\left(i,j\right)\;\;=\;\;a_{80(60)}\left(i,j\right)\;\;-\;\;a_{20}\left(i,j\right)\;\;,$$ (13)

where *a*_20_(*i*, *j*) is the ST segment amplitude, estimating the ST segment amplitude at the point J+20 ms, measured at the point *F*(*i*, *j*) + 60 ms.

#### Derivation of the KLT- and LPT-based ST segment morphologic feature-vector time series

To get the KLT- and LPT-based ST segment feature-vector time series, an *N*-dimensional (*N* = 9) KLT feature vector, $s_{K}^{{\;}^{\prime }}\left(i,j\right)$, and an *N*-dimensional LPT feature vector, $s_{L}^{{\;}^{\prime }}\left(i,j\right)$, are derived in each lead, *i*, and for each single iso-electric corrected heartbeat, *j*:

$$s_{K}^{{\;}^{\prime }}\left(i,j\right)\;=\;\Phi_{K}^{\;T}\;x\left(i,j\right)\;\;,$$ (14)

$$s_{L}^{{\;}^{\prime }}\left(i,j\right)\;=\;\Phi_{L}^{\;T}\;x\left(i,j\right)\;\;,$$ (15)

where **x**(*i, j*) is an input ST segment pattern vector composed from *M* = 32 signal samples in the interval from *F*(*i*, *j*) + 40 ms to *F*(*i*, *j*) + 160 ms, and **Φ**_K_ and **Φ**_L_ are the KLT and LPT basis function transformation matrices.

To get the final KLT and LPT feature-vector time series, **s**_K_(*i, j*) and **s**_L_(*i, j*), the coefficients of the KLT and LPT feature vectors, $s_{\;K,\;k}^{{\;}^{\prime }}\left(i,j\right)$ and $s_{\;L,\;k}^{{\;}^{\prime }}\left(i,j\right)$, are normalized according to the corresponding standard deviations, *ρ*_*k*_ and *θ*_*k*_, of the KLT and LPT expansion coefficients, respectively:

$$s_{K,k}\left(i,j\right)\;=\;\frac{s_{\;K,\;k}^{{\;}^{\prime }}\left(i,j\right)}{\rho_{k}}\;\;,\;\;\;\;\;\;k=1,2,..,N\;\;,$$ (16)

$$s_{L,k}\left(i,j\right)\;=\;\frac{s_{\;L,\;k}^{{\;}^{\prime }}\left(i,j\right)}{\theta_{k}}\;\;,\;\;\;\;\;\;k=1,2,..,N\;\;.$$ (17)

In terms of the coefficient values of the feature vectors, tiny features of the pattern vectors corresponding to higher basis functions with lower standard deviations are thus emphasized. This way, each feature of the feature vectors is normalized with the corresponding standard deviation, so that its standard deviation, *ς* is 1.

Standard deviations *ρ*_*k*_ and *θ*_*k*_ of the KLT and LPT expansion coefficients are those computed on the basis of 7,830,943 clean heart beats from the LTST DB that were used for the construction of a robust covariance matrix to derive the KLT basis functions. Table 1 shows the values of the first five standard deviations, *ρ*_*j*_ and *θ*_*j*_, of the coefficients of the KLT and LPT feature-vector time series in descending order. The magnitudes of the standard deviations computed, *ρ*_*j*_, of the KLT feature-vector coefficients are in descending order as expected. The magnitudes of standard deviations, *θ*_*j*_, of the LPT feature-vector coefficients appear to be in descending order too, as the order of the polynomials increases.

**Table 1:** Standard deviations of the coefficients of the KLT and LPT feature-vector time series.

*j*	1	2	3	4	5
*ρ*_*j*_	112.66	42.29	23.09	10.92	7.15
*θ*_*j*_	133.20	49.67	23.57	15.36	12.85

The first five standard deviations, *ρ*_*j*_ and *θ*_*j*_, of the coefficients of the KLT and LPT feature-vector time series as obtained from the LTST DB using the **Φ**_K_ and **Φ**_L_ basis functions, respectively. Values are in units (20 units = 100*μ*V).

The normalization of the coefficients also allows one to express distances between the feature vectors in terms of the Mahalanobis distance measure, *d*(*i*, *j*), between the feature vector of the *j*-th heartbeat and the feature vector of the first heartbeat as a single-dimensional compound feature useful for more comprehensive visual, as well as machine based, analysis:

$$d_{K}^{\;2}\left(i,j\right)\;=\;\sum_{k=1}^{N_{D}}\;{\left(s_{K,\;k}\left(i,j\right)\;-\;s_{\;K,\;k}\left(i,1\right)\right)}^{\;2}\;\;,$$ (18)

$$d_{\;L}^{\;2}\left(i,j\right)\;=\;\sum_{k=1}^{N_{D}}\;{\left(s_{\;L,\;k}\left(i,j\right)\;-\;s_{\;L,\;k}\left(i,1\right)\right)}^{\;2}\;\;,$$ (19)

where *s*_K, *k*_(*i*, 1) and *s*_L, *k*_(*i*, 1) are the feature vectors of the first heartbeat, and *N*_*D*_ is the Mahalanobis distance measure dimensionality.

## Results

The robust new delineation method for ST segment morphology feature extraction and transient ST segment morphology-change representation developed is capable of deriving morphologic ST segment feature-vector time series from input ECG signals. They can be used in trend plot form for representing and characterizing relevant transient ischaemic and non-ischaemic ST segment morphology categories, as well as for further automatic analysis.

**Fig 5:**
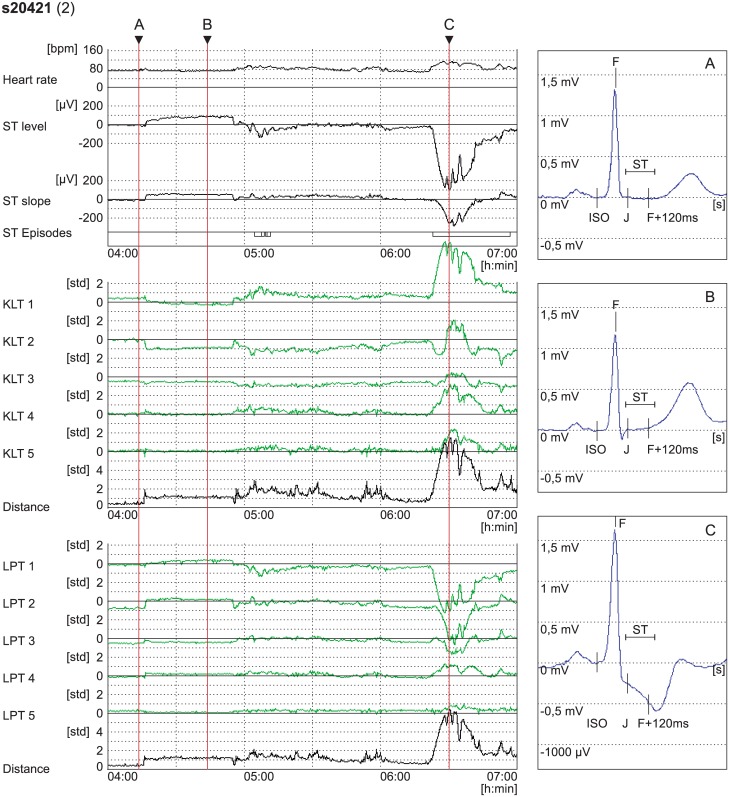
The trend plots of the derived diagnostic and morphologic feature-vector time series. The second lead of record s20421 of the LTST DB from 04:00 [h:min] to 07:00 containing axis shifts and transient ischaemic ST segment episodes. *From top to bottom*: heart rate, *h*(*j*), in [bpm]; ST segment level, *s*_l_(2, *j*), and ST segment slope, *s*_s_(2, *j*), (resolution: 100*μ*V/div); stream of human-expert annotated ischaemic ST segment episodes (long rectangles); and the first five KLT and the first five LPT ST segment coefficients, *s*_K, *k*_(2, *j*) and *s*_L, *k*_(2, *j*), with their corresponding Mahalanobis distance measures, *d*_K_(2, *j*) and *d*_L_(2, *j*), respectively, (resolution: 1*ς*/div). *At the right*: Heart beats according to markers A, B and C, prior to the axis shift [04:13:26.176], after the axis shift [04:43:30.416], and at the extrema of ischaemic ST episode [06:30:08.056] with marked interval (ST) where the KLT and LPT basis functions reside.

**Fig 6:**
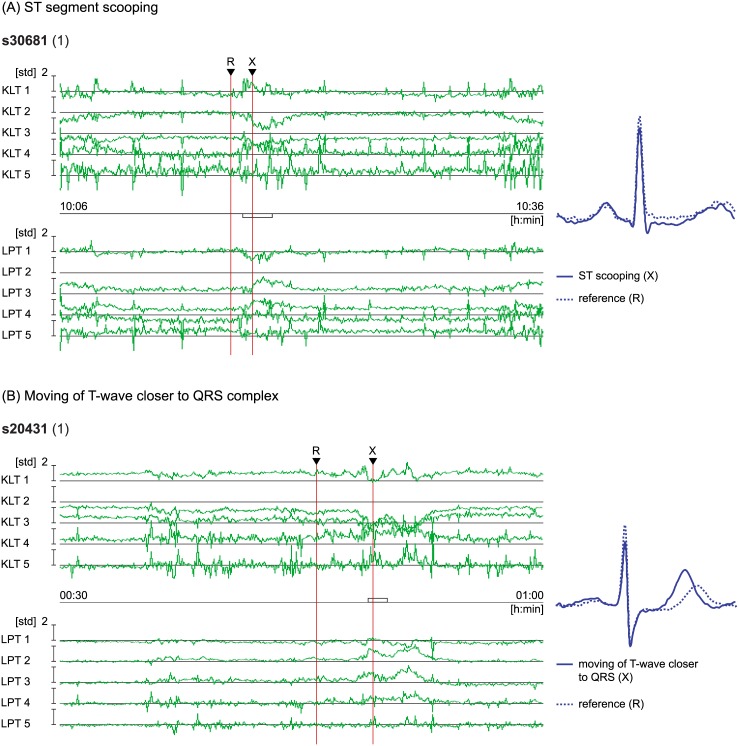
ST segment episodes in terms of the KLT and LPT feature-vector time series representation. (A) Example of ischaemic ST segment episode showing ST segment scooping (the first lead of record s30681, from 10:06 [h:min] to 10:36). (B) Example of non-ischaemic heart-rate related ST segment episode showing moving of the T wave closer to the QRS complex (the first lead of record s20431, from 00:30 to 01:00). *Upper*: The KLT feature-vector time series, **s**_K_(*i, j*). *Middle*: Human-expert annotated depressed ischaemic or elevated non-ischaemic ST segment episode. *Lower*: The LPT feature-vector time series, **s**_L_(*i, j*). *Right*: Normal reference heartbeat (dotted line) at the time marked with R with overlaid ischaemic or non-ischaemic heartbeat (solid line) at the extrema of the episode at the time marked with X.

We derived KLT- and LPT-based feature-vector time series for all records of the LTST DB. The entire LTST DB feature-vector time series collection is free and available online on PhysioNet [2] web site (https://www.physionet.org/physiobank/database/ltstdb/, doi:10.13026/C2G01T). The time series of the first nine KLT coefficients, $s_{\;K,\;k}^{{\;}^{\prime }}\left(i,j\right)$, and of the normalized KLT coefficients, *s*_*K*, k_(*i*, *j*), with the corresponding residual errors, are stored in the *.kls and *.nks files of the LTST DB (https://www.physionet.org/physiobank/database/ltstdb/kl-single-uncentralized/); while the time series of the first nine LPT coefficients, $s_{\;L,\;k}^{{\;}^{\prime }}\left(i,j\right)$, and of the normalized LPT coefficients, *s*_L, *k*_(*i*, *j*), with the corresponding residual errors, are stored in the *.loc and *.noc files of the LTST DB (https://www.physionet.org/physiobank/database/ltstdb/legendre/). Examples of trend plots of derived morphologic feature-vector time series for the selected records of the LTST DB are shown in Fig 5 and in Fig 6.

### Representation of transient ST segment morphology categories

During the annotating of the transient ST segment episodes of the LTST DB, expert cardiologists defined two classes of ST segment change episodes with the following characteristics [1]: 1) Transient ischaemic ST segment change episodes that are characterized by the typical triangular temporal pattern of ST segment level and may or may not be accompanied by a change in heart rate, when clinical information from the subject suggests ischaemia, with typical and most often ST segment morphology change categories like: depression, elevation, horizontal flattening, down-sloping, and scooping; and 2) Transient non-ischaemic ST segment change episodes that are characterized by the typical triangular temporal pattern of ST segment level and by obligatory simultaneous change in heart rate, when clinical information from the subject does not suggest ischaemia, with the typical and most frequent ST segment morphology change categories like: J-point depression with positive slope, moving of T wave into ST segment, parallel shift of ST segment compared to the reference or basal ST segment, and T wave peaking. As we present next, these categories of ST segment morphology changes can better be tracked by the ST segment feature-vector time series obtained using new KLT and LPT basis functions to derive ST segment feture-vector time series.

Fig 5 shows an example of morphologic feature-vector time series for a record from the LTST DB containing axis shifts and ischaemic ST episodes. Considering heart beats at the markers A and B, before and after the axis shift, morphology change is clearly visible, and the ST segment, in the region where basis functions reside (interval marked as ST), is elevated and with a positive slope. The latter two morphology changes can be observed in the trends of time domain diagnostic parameters and in the corresponding time series of the first and second LPT coefficient time series (LPT 1, LPT 2), but the feature-behavior mapping is less straightforward in the first and second KLT coefficient time series (KLT 1, KLT2). Plots KLT 3 and LPT 3 at markers A and C illustrate how the third coefficient of the LPT provides clearer estimations of scooping, as expected, because of its exact square-function derived shape, as opposed to the third coefficient of the KLT. Similarly, considering heartbeat C at the extrema of an ischaemic ST episode, the ST segment is depressed, down-sloped and shows negative scooping. There three morphology changes can be observed in the trends of the time series of the first, second and third LPT coefficient time series, but are less directly mapped in the KLT coefficient time series. From the trends of time domain diagnostic parameters, i.e., ST segment level and slope (the upper part of Fig 5), scooping cannot be distinguished at all.

In Fig 6 typical morphology changes during transient ischaemic and non-ischaemic ST segment episodes are shown. Fig 6A shows an example of a depressed (negative) ischaemic ST episode with ST segment scooping, while Fig 6B shows an example of elevated non-ischaemic ST episode due to the moving of the T wave closer to the QRS complex. The ST segment morphology changes correlate with the shapes of the corresponding basis functions (see Fig 4) and the morphology change category can quickly be visually determined from the trend plots. The time course of the LPT coefficient time series in example A shows depression (1st coeff.), positive slope (2nd coeff.) and positive scooping (3rd coeff.); while in example B, elevation (1st coeff.), positive slope (2nd coeff.) and positive scooping (3rd coeff.). This is consistent with the actual transient morphology change of the two episodes, and shapes and signs of the first three LPT basis functions. These significant transient morphology changes are clearly manifested and also visible in the KLT coefficient time series in both cases, but with the opposite sign of the first three KLT coefficient time series. This is due to the close similarity of the first three KLT basis functions to the constant, linear function, and square function, but with opposite sign of the three basis functions (see Fig 4).

### Evaluation of the classification between transient ischaemic and non-ischaemic ST segment episodes

Since ECG ST segment morphology delineation provides fundamental features to be used in subsequent automatic analysis, we performed an evaluation study on the classification power of the KLT- and LPT-based feature vectors, s_K_(*i, j*) and s_L_(*i, j*), to classify between the transient ischaemic and non-ischaemic ST segment episodes from the LTST DB. We set out to classify episodes of changed ST segment morphology from the entire LTST DB to the class of episodes caused by ischaemia and to the class of non-ischaemic heart-rate related episodes.

We used five established classifiers: k-Nearest Neighbors (*k*NN), Classification Tree (CT), Quadratic Discriminant Analysis (QDA), Support Vector Machines (SVM) with second-order polynomial kernel using Least Square method, and Naive Bayes Classifier (NBC) with distribution by kernel smoothing density estimate. Mathworks Matlab Statistics Toolbox algorithm implementations were used. Classification performance results are summarized in Table 2 in terms of Sensitivity, *Se* = *TP*/(*T* *P*+*FN*), Specificity, *Sp* = *TN*/(*TN*+*FP*), and Classification Accuracy, *C* *A* = (*TP*+*TN*)/(*TP*+*FN*+*TN*+*FP*), where *TP* denotes the number of true positives (ischaemic episodes classified as ischaemic), *FN* the number of false negatives (ischaemic episodes classified as non-ischaemic), *TN* the number of true negatives (non-ischaemic episodes classified as non-ischaemic), and *FP* the number of false positives (non-ischaemic episodes classified as ischaemic).

**Table 2:** Classification performance evaluation results.

		KLT			LPT		
		*Se*_KLT_(%)	*Sp*_KLT_(%)	*CA*_KLT_(%)	*Se*_LPT_(%)	*Sp*_LPT_(%)	*CA*_LPT_(%)
Coeff. 1-3	3NN	79	74	**78**	69	61	68
	4NN	76	83	77	64	65	64
	5NN	72	86	74	59	73	62
	CT	82	56	**78**	78	47	**73**
	QDA	53	85	58	45	85	53
	SVM	72	64	71	59	63	60
	NBC	71	68	70	62	67	63
Coeff. 1-5	3NN	88	82	**87**	83	75	**82**
	4NN	85	84	85	79	82	79
	5NN	82	86	83	75	84	76
	CT	87	63	83	84	59	80
	QDA	69	82	71	61	82	65
	SVM	79	75	78	77	74	76
	NBC	81	69	79	70	70	70
Coeff. 1-8	3NN	91	85	**90**	85	72	**82**
	4NN	89	85	88	82	79	81
	5NN	87	87	87	79	80	79
	CT	90	68	86	86	56	81
	QDA	90	53	84	90	40	81
	SVM	81	76	81	81	74	79
	NBC	83	73	81	74	67	73

Classification between ischaemic and non-ischaemic heart-rate related ST segment episodes using *k*-Nearest Neighbors (*k*NN) with *k* = 3 (3NN), *k* = 4 (4NN), and *k* = 5 (5NN), Classification Tree (CT), Quadratic Discriminant Analysis (QDA), Support Vector Machines (SVM), and Naive Bayes Classifier (NBC) using the KLT and LPT feature vectors. *Se*—Sensitivity, *Sp*—Specificity, *C* *A*—Classification Accuracy. The highest classification accuracy for each group of coefficients is bold.

Each episode of the LTST DB annotated in each single ECG lead according to the annotation protocol B [1] was represented by a mean feature vector of the KLT and a mean feature vector of the LPT coefficients, derived from an interval of 20 seconds around the episode extreme. Classification performance evaluation results are shown in Table 2. Classification power was tested using three different feature transformation-coefficient subsets: coefficients 1-3, coefficients 1-5 and coefficients 1-8. These feature vectors were used as input data for classification performance evaluation by 10-fold cross-validation with 10 repetitions. Classification was performed separately with the KLT and LPT feature vectors. The resulting input data consisted of 1130 instances of ischaemic ST segment episodes and 234 instances of non-ischaemic heart-rate related ST segment episodes. The highest classification performance was obtained using the *k*NN, *k* = 3, the KLT coefficients 1-8, *Se*_KLT_ = 91%, *Sp*_KLT_ = 85%, and *CA*_KLT_ = 90%.

## Discussion and conclusions

The Legendre polynomials as basis functions of the transformation of the ECG ST segment proved to be convenient for the purposes of feature extraction and shape representation because of their simple generation process and orthogonality. Visual examination confirmed that the new LPT approach based on the Legendre polynomials is a valid representation of ST segment morphology. It has the unique additional benefit of direct insight into the clinically important typical time domain ST segment morphology changes. Typical time domain characteristics of transient ST segment morphology changes like elevation or depression, up- or down-sloping, and scooping are clearly visible in feature-vector time series (Figs 5 and 6). The shapes of the first three Legendre polynomials (a constant, linear and square function) precisely correspond to these characteristics and thus provide accurate extraction of the desired features.

While trends of time domain diagnostic parameters show ST segment level and slope changes of ischaemic ST episodes as measured at a single point (J + 80(60) ms) only, time series of the KLT and LPT feature-vector coefficients offer clearer insight into the categories of transient ST segment morphology changes. This is especially true for the subtle scooping of ST segment (see Figs 5 and 6), which can not be efficiently measured in time domain. The third coefficient of the LPT produces clear deviations consistent with visually detectable shapes. Clinicians can easily examine important features by visual observation of the LPT feature-vector time series trends spanning several hours on a single display. This is significantly faster than evaluating ST segment morphology changes on the level of individual heart beats or examining feature-vector time series without clearly understandable time domain meaning. On the other hand, the developed delineation method, especially using the KLT, offers a more comprehensive estimation of ST segment features for automated systems in contrast to the traditional time domain measurements (ST segment level and slope at fixed points in the ST segment) since the orthogonal transformation based techniques extract information of morphology from the entire ST segment. Another advantage is that detection of the J point can be omitted.

The results of the evaluation study on classification performance (Table 2) show the potential of the feature vectors based on the new KLT and LPT basis functions for classification between ischaemic and non-ischaemic ST segment episodes. Besides assessing classification performance in distinguishing ischaemic from non-ischaemic ST segment episodes, our goal was also to assess classification performance of the KLT compared to the LPT. Classifiers used in this study perform slightly better if using the new KLT feature vectors than using the new LPT feature vectors (*Se*_KLT_ = 91%, *Sp*_KLT_ = 85%, *CA*_KLT_ = 90%; compared to *Se*_LPT_ = 85%, *Sp*_LPT_ = 72%, *CA*_LPT_ = 82%; for the best-performing classifier *k*NN, *k* = 3, and using the best performing feature subset, coefficients 1-8). This was expected, as the LPT is based on the Legendre polynomials and is not fitted to any “training” data in comparison to the KLT. In spite this the classification performances are still comparable.

Related studies on the classification between ischaemic and non-ischaemic ST segment episodes employed a variety of other approaches. A decision tree based classification between ischaemic and non-ischaemic ST segment episodes of the LTST DB was performed in [18]. In this study, compound features like heart rate, Legendre polynomial coefficients, and the Mahalanobis distance of the QRS complex KLT coefficients were used. These compound features were actually derived as differences between features of pre-episode onset, pre-episode offset, and episode extreme. Classification performances achieved on the entire set of ischaemic and non-ischaemic ST segment episodes of the LTST DB were 98.4% (*Se*) and 85.9% (*Sp*). Using the bootstrap method to assess the robustness of the performance statistics and to predict the real-world performance of the approach, the 5% confidence limits achieved were 97.8% (*Se*) and 81.5% (*Sp*). A similar discriminant analysis based approach [19] in the classification of ischaemic and non-ischaemic ST segment episodes of the LTST DB used compound features derived as differences between features of pre-episode onset, pre-episode offset, and episode extreme as well. The features used were heart rate, QT interval, ST segment level, and QRS complex and ST segment KLT coefficients. The classification performances achieved using leave-one-out cross-validation estimation were 84.5% (*Se*) and 86.6% (*Sp*). Yet another study [20] conducted a genetic algorithm-based selection to identify the nine, out of 35, most relevant features to classify ischaemic and non-ishaemic ST segment episodes of the LTST DB. The selected features were single features like the low to high frequency ratio (LF/HF ratio) of heart rate ST segment level, and the root mean square of the ST segment shape change, and compound features (differences in features from pre-episode onset and pre-episode offset) like heart rate, ST segment slope, R wave up-slope, root mean square of the QRS complex shape change, and the Mahalanobis distances of the QRS complex and ST segment KLT coefficients. Ischaemic and non-ischaemic ST segment episodes from the LTST DB were separated into training and testing sets. The performances obtained from the testing set using the Relevance vector machine (a special case of a sparse Bayesian learning algorithm) were 88.7% (*Se*) and 86.8% (*Sp*).

Our study intentionally used non-compound features and those based solely on the KLT- or LPT-based feature vectors. Our goals were to assess the classification performance to classify between ischaemic and non-ischaemic ST segment episodes, and to assess the classification performance of the KLT-based feature vectors as opposed to the LPT-based feature vectors. To assess the classification performance we used 10-fold cross-validation with 10 repetitions. In comparison to [18–20] we also tested a variety of classifiers (kNN, CT, QDA, SVM, NBC). The *k*-Nearest Neighbors classifier yielded the highest classification performances if using the KLT-based feature vectors, *Se*_KLT_ = 91% and *Sp*_KLT_ = 85%. The classification performances achieved are quite comparable to the performances obtained in [18], and higher than those obtained in [19, 20]. The Mahalanobis distances of the QRS complex and ST segment KLT coefficients in [18, 20] and the QRS complex and ST segment KLT coefficients in [19, 20] were derived using the KLT basis functions [7] from the ESC DB. These basis functions span over two ECG leads (for these ST segment KLT basis functions see Fig 3). Consequently, we may expect that the extracted features in terms of the KLT coefficients will less accurately emphasize the presence of ischaemia, since ischaemic ST segment change shapes may be significant in one ECG lead, but less significant, or even absent, in the other ECG lead. One of the advances of the proposed method lies in the fact that the newly developed KLT basis functions, and the LPT basis functions, span over a single ECG lead, thus the extracted features will more accurately emphasize the presence, or not, of ischaemic ST segment shape changes. The next advance of the proposed method lies in the reduced set of features (the KLT- and LPT-based feature vectors only) that yield high classification performance, the only prerequisite is a stable fiducial point for each heartbeat. Furthermore, extracting the morphologic features of ST segments exclusively in terms of the KLT or LPT feature vectors is more robust in the sense that they are less susceptible to ECG signal variability and noise. Another advance of the proposed method are non-compound features that are extracted only at the extrema of ischaemic and non-ischaemic ST segment episodes. There is no need for the accurate detection of the beginnings of the episodes, which is difficult.

The LPT offers an additional asset of immediate insight into the type and shape of ST segment change in time domain. This fact indicates possibilities for the development of new clinical diagnostic criteria for the reliable visual detection of transient ischemia using the LPT. Consequently, significantly lower rates of erroneously estimated ST segment features can be expected.

As for the task of the delineation of significant transient ST segment morphology changes from the entire ST segment, we conclude that the LPT basis functions provide higher accuracy in the representation of transient ST segment morphology categories, while the new KLT basis functions provide higher classification accuracy between ischaemic and non-ischaemic ST segment episodes, offering future applications such as new automated transient ischaemia detection systems.
